# Secular Trends in Growth and Nutritional Outcomes of Children under Five Years Old in Xiamen, China

**DOI:** 10.3390/ijerph13111104

**Published:** 2016-11-09

**Authors:** Jing Chen, Wei Chen, Guozhang Zeng, Guimei Li

**Affiliations:** 1 Department of Pediatrics, Shandong Provincial Hospital Affiliated to Shandong University, Jinan 250014, China; lgmusa05@hotmail.com; 2 Department of Child Health, Maternal and Child Health Care Hospital, Xiamen 361003, Fujian, China; kunlichen@163.com; 3 Department of Safety and Security, Xiamen University, Xiamen 361003, Fujian, China; chenw024@163.com

**Keywords:** children under five, China, obesity, overweight, under-nutrition

## Abstract

The purpose of this study was to examine secular changes in growth and nutritional outcomes of Chinese urban children under five years old, in 2009, 2012, and 2015. Cluster random sampling methods were used to select children under five years old in Xiamen, one of five special economic zones in China. Subjects (N = 71,229) under five years old (39,413 boys, 31,816 girls) were examined at three different times (22,576 in 2009, 24,816 in 2012, and 23,837 in 2015). Significant differences in the boys’ height and weight (*p* < 0.05) were found across the three time points; subjects in the 2015 sample were the heaviest and tallest, and they had the highest BMI (2009 < 2015; 2012 < 2015). Among the girls, those in the 2015 sample were similar to the boys (2009 < 2012; 2012 < 2015). In general, similar patterns were observed when mean values were analyzed by age. An increasing trend was found in the prevalence of overweight and obesity and a decreasing trend for stunting, underweight, and wasting. The results revealed that the burden of childhood under- and over-nutrition might constitute a public health concern in modern China.

## 1. Introduction

In many developing countries, child malnutrition is a long-term and persistent public health problem [1], which may lead to underweight, wasting, or stunting. Although global data show that the prevalence of children’s stunting and wasting has been falling [1], this unfortunate situation remains a public health challenge, especially in developing countries. On a global scale, for example, 15.1% of children less than five years old were underweight, 24.7% showed stunting, and 8% exhibited wasting (including severe wasting in 3%) in 2012; Furthermore, 92% of stunted children, 96% of underweight children, and 97% of wasted children live in Africa and Asia [2].

Although malnutrition persists in some places around the world, the prevalence of pediatric overweight and obesity is increasing worldwide [3]. The increasing incidence of obesity among young people is considered a serious public health problem because it is closely related to adult obesity and its complications. At present, the incidence of obesity continues to increase rapidly in most countries. From the 1970s to the late 1990s, the incidence of overweight and obesity of school-age children increased two to three times in several economically developed countries [4].

Given the rapid growth of the national economy, the nutritional outcomes of the Chinese people have improved considerably in the past dozen years [5]. Xiamen is one of five special economic zones of China and the major economic zone. Over the past ten years, the rapid development of the economy in Xiamen has led to substantial changes in the way of life of its people, which may also have affected the prevalence of malnutrition and over-nutrition in children. However, no relevant research studies about children’s nutrition or health surveys have been conducted in Xiamen. Therefore, in this study, we aimed to provide data on the prevalence of underweight, stunting, wasting, overweight, and obesity among children <5 years old in Xiamen between 2009 and 2015. This study was intended to provide the latest evidence supporting the government’s policies to improve children's nutrition.

## 2. Subjects and Methods

### 2.1. Subjects and Sampling

The study was a cross-sectional survey of anthropometric measurements of children <5 years of age. Briefly, its goals were to describe the patterns of variability in the growth and development of children <5 years of age, and to understand the role of genetic and environmental factors in the variability of these indicators in this population. Data were collected at three time points: 2009, 2012, and 2015.

Cluster random sampling and the stratified sampling methods were used to select children under five years old from 1 April–30 June at each time point in Xiamen, China. The subjects were selected from nine community health service centers in three districts of Xiamen, and children without a signed consent form, with physical handicaps, chronic diseases, or psychological disorders were excluded from the study. The subjects in the final sample were distributed as follows: 22,576 subjects in 2009 (12,574 boys, 10,002 girls); 24,816 subjects in 2012 (13,586 boys, 11,230 girls); and 23,837 subjects in 2015 (13,253 boys, 10,584 girls). The total sample consisted of 71,229 subjects (39,413 boys, 31,816 girls; Table 1). The study was approved by the Human Research Ethics Committee of Xiamen Maternal and Child Health Care Hospital (KY-2016002).

### 2.2. Anthropometry

Information, including the child’s age, sex, and other data (family situation, birth history, feeding history, and so on) was obtained by interviewing their caregivers. The same measurement protocol was used for the three data collections [6]. Anthropometric measurements, including height and weight, were used to assess the nutritional outcomes. Height and weight were measured using the same personnel with the same training in order to minimize measurement error. Height was measured using a standard calibrated board (0.1 cm; Jiangsu Nantong Weighing Apparatus Factory, Nantong, China), and a standard calibrated balance scale (0.1 kg; Bailida electronic scale; Shanghai Instrument Technology Company, Shanghai, China) was used to measure weight. Weight was measured with the subject wearing light clothes and without shoes. Height was measured with the shoes off, feet together, and head in the horizontal plane. The length of children under two years was measured in a lying posture. Body mass index (BMI) was calculated using weight and height (kg/m^2^) measurements.

### 2.3. Diagnostic Criteria

Nutritional status was determined according to cut-offs suggested by the World Health Organization (WHO) expert committee [7]. In detail, diagnosis standards of under- and over-nutrition are as follows according to the 2006 WHO Child Growth Standards: (1) underweight: weight-for-age below two standard deviations (SD) from the median value based on the child’s specific age and sex; (2) stunted: height-for-age below two standard deviations (SD) from the median value based on the child’s specific age and sex; (3) wasted: weight-for-height below two standard deviations (SD) from the median value based on the child’s specific age and sex; (4) overweight: BMI-for-age ≥P85 and <P95; and (6) obese: BMI for age ≥P95 [8,9].

### 2.4. Statistical Analyses

Basic descriptive statistics were computed. To compare the means of multiple groups, we used univariate ANOVA with the F statistic or Welch correction for heteroscedasticity. For multiple comparisons, we used the least significant difference (LSD) test or Dunnetts’s C test. The prevalence of stunting, underweight, wasting, and overweight/obesity was determined in relation to age and sex. Malnutrition and overweight/obesity rates were compared between groups using the χ^2^ test. Differences in the prevalence of nutritional outcomes across the years were examined using the χ^2^ test. The SPSS statistical software package version 13.0 (SPSS Inc., Chicago, IL, USA) was used for all analyses. The significance level, *p* < 0.05, was used for all analyses.

## 3. Results

The results of the ANCOVA (mean ± SD, F, and *p*-values) for height, weight, and BMI in boys and girls under five years old by year of evaluation are presented in Table 2. Among the boys, height and weight of the subjects from 2015 were the heaviest and the tallest. The highest BMI was observed in 2015, with increases found in 2009–2015. The results of the girls were similar to the boys.

Table 3 shows the ANCOVA results for the mean differences across the three years for each variable by sex and age. In summary, increases in weight and BMI were found in each age group of boys and girls from 2009 to 2015, with the subjects from 2015 being heavier and having the highest BMI; however, significant differences were not found, at age 3– and 4–5 years of both sexes (*p* > 0.05). With respect to height, generally, the children evaluated in 2015 had the highest values compared with those evaluated in 2009; an increase in stature was detected in both sexes.

**Figure 1:**
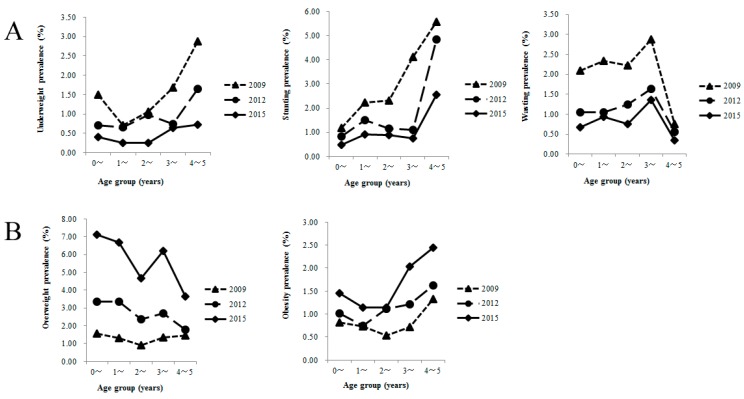
(**A**) Prevalence of underweight, stunted, and wasted children aged 0–5 years old in Xiamen; (**B**) Prevalence of overweight and obese children aged 0–5 years old in Xiamen.

Changes in nutritional outcomes across the three time points are summarized in Table 4. In general, an increasing trend in the past seven years was observed in the prevalence of overweight and obesity, whereas a decreasing trend was found for underweight, stunting, and wasting. The prevalence of overweight decreased with age, and the prevalence of obesity increased and peaked at age 4–5 years. Underweight and stunting occurred mostly at age 4–5 years, whereas wasting occurred mainly at age 3–4 years (Figure 1).

## 4. Discussion

The purpose of this study was to examine secular changes in growth and nutritional outcomes of children less than five years old in Xiamen, China, one of five special economic zones, in 2009, 2012, and 2015. In general, a positive secular trend was observed in height, weight, and BMI mean values among Chinese children aged 0–5 years. This phenomenon not only reflected faster growth in childhood, earlier puberty, and steady increments in adult height, but also dramatic changes in body shape [10]. This might be associated with changes in the population, specifically general improvements in their lifestyle, health, nutritional, and economic conditions during this period. However, there were also negative effects, including higher BMI, which can lead to hypertension, hyperglycemia, type 2 diabetes, and other adulthood diseases that may occur earlier in childhood and adolescence [11,12].

China is experiencing a nutrition transition [13,14]. The Chinese Ministry of Health have reported that the prevalence of stunting, wasting, and being underweight was 9.9%, 2.3%, and 3.6% (2010) [15]; the National Program of Action for Child Development in China (2011–2020) has pointed out that the goals for the prevalence of stunting and underweight of children under five are below 7% and 5%, respectively, in 2020 [16]. Our study showed a decline in the prevalence of stunting, wasting, and being underweight between 2009 and 2015 in Xiamen, which is significantly lower than the national level and beyond the goals of 2020. Meanwhile, the prevalence of under-nutrition in Xiamen was also significantly lower than the neighboring Asian countries, and partially superior to the levels found in some developed countries in the West [17,18,19]. For example, the prevalence of stunting, wasting, and being underweight was 30.3%, 7.9%, and 19.9% in the Philippines (2013–2014); 38.7%, 15.1%, and 29.4% in India (2013–2014); 2.1%, 0.5%, and 0.5% in the United States (2011–2012); and 2%, 0%, and 0.2% in Australia (2012) [17]. The Xiamen municipal government has vigorously promoted all sorts of nutrition improvement measures in recent years, including improved maternal nutrition, an early initiation of breastfeeding, exclusive breastfeeding for the first six months of life, and timely, adequate, safe, and appropriate complementary feeding. The government’s intervention programs targeted to improve childhood nutrition status may have played an important part in this change.

Our cross-sectional data suggest that the overall prevalence of obesity and overweight in children increased significantly between 2009 and 2015 in the Xiamen area. In contrast, no recent increases in the prevalence of childhood obesity have been found in Sweden [20] or Australia [21]. In France [22] and Greece [23], the prevalence of childhood obesity rates even fell in recent years. During the past twenty years, an increasing incidence of childhood obesity in these developed countries has prompted the governments to plan healthcare interventions to prevent childhood obesity. Various public health actions for improving dietary habits and physical activity in children have been implemented. For example, “Nutrition, Prevention and Health for children and teenagers in Aquitaine” launched in France between 2004 and 2008 had improved eating habits of children. Intake of light afternoon meals had increased while snacking in the morning and nibbling had decreased [24]. These changes have played a very important role in combating childhood obesity and led to stabilizing or decreasing the prevalence of overweight and obesity in recent years.

Our research shows that the changes in the prevalence of malnutrition and obesity were age-specific in Xiamen, China. Underweight and stunting occurred mainly in 4–5 year olds (the oldest age group in our cohort), and wasting occurred mainly in 3–4 year olds. In general, malnutrition peaked mainly in the children aged 3–5 years old. The shift in the peak age of child malnutrition might be related to the government’s policy in recent years, of vigorously promoting breastfeeding and adding supplementary food based on scientific evidence and reason. This finding suggests that the critical period for preventing and treating malnutrition should be the preschool period. Preschool children’s nutrition is dependent mainly on dietary intake. Therefore, whether dietary composition is scientifically sound and appropriate has an important influence on the health of children. From the perspective of the population’s distribution, the highest prevalence of overweight children was found in the 0–1 year olds (the youngest age group in our cohort), then it decreased with age. However, this result is in contrast to previous research reports that the prevalence of overweight increased continuously with age [25,26]. The highest prevalence of obesity was observed in the 4–5 year olds, which suggests that the prevention and treatment of childhood obesity, especially in preschoolers, is a dire necessity in China. The underlying cause of these age and gender-specific patterns is largely unknown.

In our study, we found that both obesity and being overweight were higher in boys than in girls across the three time points (2009, 2012, and 2015). This phenomenon is similar to some European countries [27,28], but it was not observed in Jordan [29] or Hungary [30]. The higher prevalence observed in boys might be related to China’s traditional culture, different views of what body shape is most aesthetically pleasing for boys versus girls, and different requirements for their stature [31]. However, there were no significant differences between the genders in the prevalence of stunting, wasting, and being underweight in 2009, 2012, and 2015.

Notwithstanding the relevance of the present results, this study has several limitations. First, data for this study were obtained from three independent cross-sectional surveys rather than from a longitudinal cohort study, thereby preventing further assessment of the cohort and time effects. Second, the sample’s restriction to children in Xiamen did not allow us to observe these trends in the rural areas of China. Third, the children’s nutritional habits and/or physical activity levels and patterns were not objectively measured, which would have been very useful for interpreting the results. One recent Chinese study in Shantou city, another economic zone in China similar to Xiamen, indicated that inappropriate dietary intakes and lack of physical activity levels might lead to the imbalance of energy intake and consumption and associate with higher prevalence of overweight and obesity [32]. However, the sample size we surveyed is as large as 71,229 urban children under five years old, and the self-description of dietary energy intake and physical activity levels were difficult to assess accurately, even if a unified method of measurement was informed. Meanwhile it must be understood that collecting the data systematically and objectively would have been a very difficult task over a nearly 10-year period.

## 5. Conclusions

Significant positive changes in growth and nutritional outcomes were observed among children from Xiamen who were <5 years old, from 2009 to 2015, which are associated with societal and economic conditions, standard of living, and caregivers’ feeding knowledge [17]. This finding suggests that positive changes in growth and nutritional outcomes are increasing in the urban areas of China. However, at the same time, BMI has increased dramatically during the past seven years, which indicates that the burden of childhood over-nutrition constitutes a public health concern in modern China and requires more attention. Overweight and obesity have become major threats to children’s health. Therefore, we should undertake comprehensive efforts to prevent and control children’s obesity by implementing regular monitoring, nutrition education, aerobic exercise, and healthy eating behaviors.

## Figures and Tables

**Table 1:** Sample size of each age and sex group by year of evaluation (B = boys; G = girls).

Age	2009			2012			2015			Total
	B	G	Total	B	G	Total	B	G	Total	
0~	2514	2105	4619	2672	2293	4965	2549	2076	4625	14,209
1~	2456	1936	4392	2751	2247	4998	2550	2224	4774	14,164
2~	2598	2035	4633	2643	2310	4953	2600	2150	4750	14,336
3~	2482	1974	4456	2720	2260	4980	2834	2054	4888	14,324
4–5	2524	1952	4476	2800	2120	4920	2720	2080	4800	14,196
Total	12,574	10,002	22,576	13,586	11,230	24,816	13,253	10,584	23,837	71,229

**Table 2:** Age-adjusted means and standard deviations for height, weight, and BMI in boys and girls (0–5 years old) by year of evaluation.

Variable	2009	2012	2015	F	*p*-Value
	Mean ± SD	Mean ± SD	Mean ± SD		
Boys					
Height	69.2 ± 11.3	73.7 ± 13.4	77.2 ± 13.5	1772.19	<0.001 ^1,2,3^
Weight	8.3 ± 2.8	9.3 ± 3.3	9.9 ± 3.3	1323.78	<0.001 ^1,2,3^
BMI	16.3 ± 1.6	16.6 ± 1.7	16.8 ± 1.6	399.30	<0.001 ^1,2,3^
Girls					
Height	67.9 ± 11.3	72.1 ± 13.5	74.7 ± 13.6	1717.36	<0.001 ^1,2,3^
Weight	7.7 ± 2.7	8.6 ± 3.2	9.1 ± 3.2	1298.63	<0.001 ^1,2,3^
BMI	15.9 ± 1.5	16.1 ± 1.6	16.2 ± 1.6	515.96	<0.001 ^1,2,3^

^1^ Significant difference between 2009 vs., 2012. ^2^ Significant difference between 2009 vs. 2015. ^3^ Significant difference between 2012 vs. 2015.

**Table 3:** Means and standard deviations for height, weight, and BMI in boys and girls by months of age and year of evaluation (ANCOVA results).

Variable	Height (Mean ± SD)				Weight (Mean ± SD)				BMI (Mean ± SD)			
	2009	2012	2015	Pairwise Comparisons	2009	2012	2015	Pairwise Comparisons	2009	2012	2015	Pairwise Comparisons
Boys				(only when F has a *p* < 0.05)								
0~	56.3 ± 2.3	56.4 ± 2.2	56.5 ± 2.2	09 = 12 09 < 15 12 = 15	4.9 ± 0.6	4.9 ± 0.7	5.0 ± 0.6	09 = 12 09 < 15 12 < 15	15.5 ± 1.3	15.5 ± 1.3	15.6 ± 1.3	09 = 12 09 < 15 12 < 15
2~	63.1 ± 3.6	63.1 ± 3.6	63.5 ± 3.5	09 = 12 09 < 15 12 < 15	6.9 ± 1.1	6.9 ± 1.1	7.1 ± 1.1	09 = 12 09 < 15 12 < 15	17.1 ± 1.7	17.2 ± 1.6	17.4 ± 1.6	09 < 12 09 < 15 12 < 15
6~	71.0 ± 3.5	71.1 ± 3.5	71.4 ± 3.5	09 = 12 09 < 15 12 < 15	8.7 ± 1.2	8.8 ± 1.2	9.0 ± 1.1	09 < 12 09 < 15 12 < 15	17.2 ± 1.5	17.4 ± 1.6	17.6 ± 1.5	09 < 12 09 < 15 12 < 15
12~	80.4 ± 4.7	83.0 ± 6.2	84.7 ± 6.8	09 < 12 09 < 15 12 < 15	10.9 ± 1.5	11.4 ± 1.8	11.5 ± 1.9	09 < 12 09 < 15 12 < 15	16.0 ± 1.3	16.5 ± 1.3	16.8 ± 1.4	09 < 12 09 < 15 12 < 15
24~	90.8 ± 3.9	90.9 ± 4.1	91.4 ± 4.3	09 = 12 09 < 15 12 < 15	12.9 ± 1.7	13.1 ± 1.7	13.2 ± 1.7	09 = 12 09 < 15 12 < 15	15.6 ± 1.2	15.7 ± 1.3	16.0 ± 1.3	09 < 12 09 < 15 12 < 15
36~	97.9 ± 4.2	98.1 ± 3.4	98.3 ± 4.3	—	14.8 ± 2.0	14.9 ± 1.6	15.0 ± 2.1	—	15.4 ± 1.3	15.4 ± 1.3	15.4 ± 1.4	—
48~60	103.7 ± 4.3	105.1 ± 4.5	105.1 ± 4.4	—	16.4 ± 2.8	16.7 ± 2.2	16.8 ± 2.4	—	15.1 ± 1.3	15.1 ± 1.4	15.2 ± 1.1	—
Girls												
0~	55.1 ± 2.2	55.9 ± 2.4	56.2 ± 2.1	09 < 12 09 < 15 12 < 15	4.6 ± 0.6	4.8 ± 0.7	4.9 ± 0.6	09 < 12 09 < 15 12 < 15	14.9 ± 1.2	15.3 ± 1.4	15.5 ± 1.3	09 < 12 09 < 15 12 < 15
2~	59.4 ± 3.5	61.4 ± 3.4	61.6 ± 3.4	09 < 12 09 < 15 12 < 15	5.8 ± 1.0	6.2 ± 1.0	6.3 ± 1.0	09 < 12 09 < 15 12 < 15	16.2 ± 1.6	16.4 ± 1.6	16.6 ± 1.6	09 < 12 09 < 15 12 < 15
6~	69.2 ± 3.4	70.6 ± 3.9	70.7 ± 3.2	09 < 12 09 < 15 12 < 15	8.0 ± 1.1	8.4 ± 1.3	8.9 ± 1.1	09 < 12 09 < 15 12 < 15	16.7 ± 1.5	16.9 ± 1.5	17.7 ± 1.6	09 < 12 09 < 15 12 < 15
12~	78.7 ± 4.5	81.8 ± 6.2	82.9 ± 6.9	09 < 12 09 < 15 12 < 15	10.1 ± 1.3	10.7 ± 1.7	10.9 ± 1.9	09 < 12 09 < 15 12 < 15	15.7 ± 1.3	16.0 ± 1.3	16.3 ± 1.3	09 < 12 09 < 15 12 < 15
24~	89.4 ± 3.7	89.6 ± 4.1	90.2 ± 4.4	09 = 12 09 < 15 12 < 15	12.4 ± 1.6	12.5 ± 1.5	12.6 ± 1.7	09 = 12 09 < 15 12 = 15	15.3 ± 1.2	15.4 ± 1.2	15.6 ± 1.3	09 = 12 09 < 15 12 < 15
36~	96.1 ± 4.3	96.8 ± 4.2	96.8 ± 4.4	—	14.2 ± 1.9	14.3 ± 1.9	14.3 ± 1.9	—	15.1 ± 1.2	15.2 ± 1.3	15.4 ± 1.1	—
48~60	104.1 ± 5.1	104.3 ± 4.8	104.5 ± 4.3	—	15.3 ± 2.6	16.3 ± 2.7	16.5 ± 2.3	—	14.1 ± 1.4	14.9 ± 1.6	15.0 ± 1.4	—

**Table 4:** Prevalence of nutritional indicators by sex and year of evaluation and differences in prevalence between years of evaluation by sex.

Variable	2009		2012		2015			
	N	%	N	%	N	%	Trend	*p*-Value
Boys								
Underweight	157	1.25	109	0.80	48	0.36	Decreasing	<0.001
Stunted	357	2.84	197	1.45	107	0.81	Decreasing	<0.001
Wasted	204	1.62	171	1.26	127	0.96	Decreasing	<0.001
Overweight	185	1.47	440	3.24	857	6.47	Increasing	<0.001
Obese	113	0.90	136	1.00	197	1.49	Increasing	<0.001
Girls								
Underweight	120	1.20	107	0.95	46	0.43	Decreasing	<0.001
Stunted	267	2.67	130	1.16	72	0.68	Decreasing	<0.001
Wasted	144	1.44	107	0.95	59	0.56	Decreasing	<0.001
Overweight	99	0.99	314	2.80	646	6.10	Increasing	<0.001
Obese	83	0.83	92	0.82	135	1.28	Increasing	<0.001
